# Guanylyl cyclase C ameliorates visceral pain: an unsuspected link

**DOI:** 10.1172/JCI166703

**Published:** 2023-02-15

**Authors:** Rodger A. Liddle

**Affiliations:** Department of Medicine, Duke University and Veterans Affairs Health Care System, Durham, North Carolina, USA.

## Abstract

Visceral pain associated with irritable bowel syndrome afflicts 15% of the US population. Although treatments are limited, guanylyl cyclase C (GUCY2C) agonists alleviate pain and constipation. Until now, it was assumed that the activation of GUCY2C and production of cGMP in enterocytes stimulated fluid secretion and reduced visceral sensation. The recent discovery that a subtype of enteroendocrine cells (EECs) known as neuropod cells synapse with submucosal neurons unveiled a pathway for communicating gut signals to the nervous system. In this issue of the *JCI*, Barton et al. report that GUCY2C is enriched in neuropod cells and is involved with sensory nerve firing. Selective deletion of GUCY2C in mouse models suggests that defective GUCY2C neuropod–cell signaling underlies visceral pain. These studies introduce possibilities for dissociating the secretory and analgesic effects of GUCY2C agonism. Although further work remains, unveiling the role of neuropod cells is a major step in understanding visceral pain.

## Unexplained visceral pain

**Figure 1:**
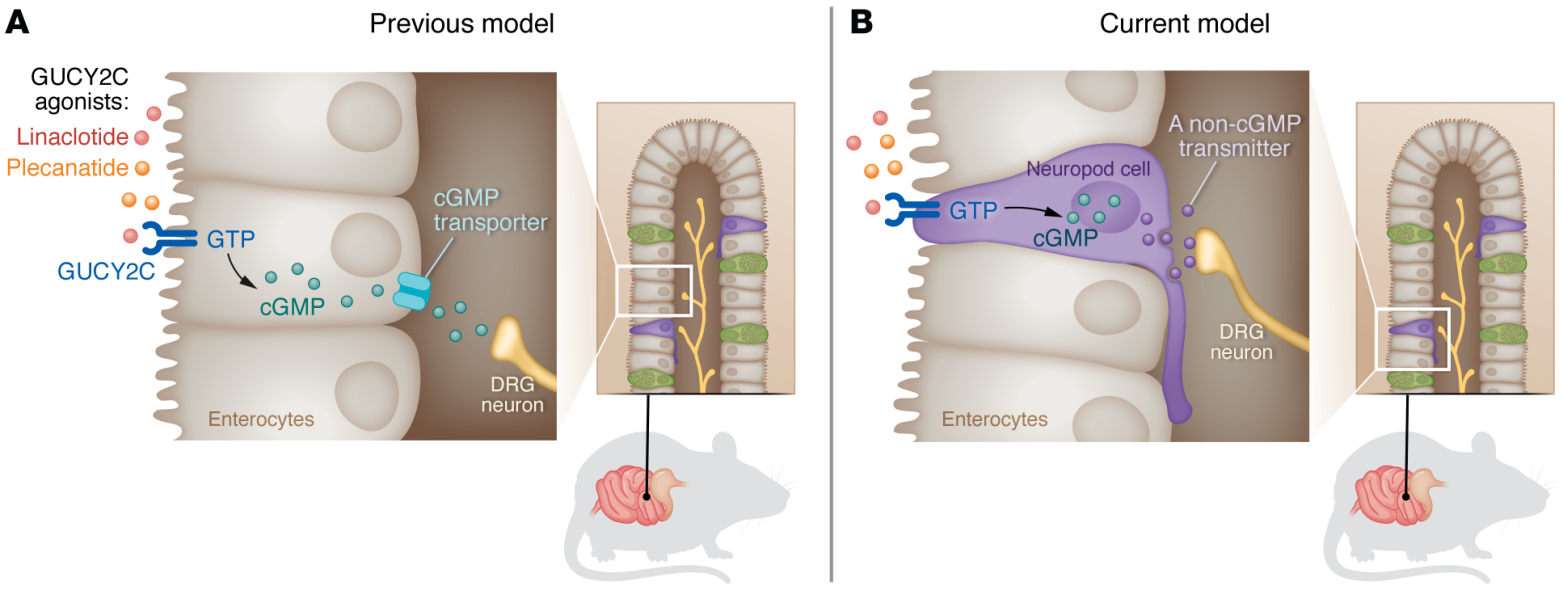
An updated model for the regulation of visceral pain involves neuropod GUCY2C. (**A**) Previously, it was believed that GUCY2C agonists, such as linaclotide and plecanatide, modulated visceral pain by activating GUCY2C on enterocytes to generate cGMP. The prior model proposed that cGMP released from the basal surface of enterocytes diffused to submucosal neurons to diminish neuronal firing. (**B**) Barton, et al. (12), demonstrated that GUCY2C agonists act on neuropod cells, which are connected to sensory neurons to ameliorate visceral pain by a non-cGMP mechanism.

It has been estimated that up to 50% of visits to a gastroenterologist are for evaluation and treatment of unexplained visceral pain, commonly referred to as irritable bowel syndrome (IBS) (1, 2). IBS is a constellation of chronic symptoms including abdominal pain associated with alterations in stool frequency and consistency such as diarrhea and/or constipation (3). The pathophysiological basis of IBS is unknown, but heightened visceral nociception has been demonstrated in clinical investigations (4). Historically, treatments for IBS have focused on symptom management; only recently have specific pharmacological therapeutics been directed at molecular targets to ameliorate symptoms. Among the most notable examples have been drugs that activate the receptor/enzyme guanylyl cyclase C (GUCY2C), a product of the *GUCY2C* gene on intestinal mucosal cells and stimulate intestinal fluid secretion and motility to treat constipation-predominant IBS. GUCY2C is expressed on the apical surface of enterocytes throughout the intestine, and, upon ligand binding, converts guanosine triphosphate (GTP) to cyclic guanosine monophosphate (cGMP). cGMP increases intestinal fluid secretion by (i) activating cystic fibrosis transmembrane conductance (CFTR) and (ii) inhibiting the apical Na^+^/H^+^ exchanger 3 (NHE3). During the development of GUCY2C agonist drugs — e.g., linaclotide and plecanatide — for the treatment of constipation, it was noted that they also reduced visceral pain in both animal models and humans. Since cGMP is released from enterocytes and cGMP reduces the firing of nociceptive neurons, it was naturally assumed that both the secretory and nociceptive effects of GUCY2C agonists were mediated by enterocytes (Figure 1). However, a problem remained.

Although cGMP is indeed released from enterocytes following GUCY2C activation, apical secretion greatly exceeds basolateral release (5). Thus, the concentrations of cGMP in the gut lumen are much higher than in the submucosa where neurons reside. In fact, using microdialysis techniques, one study demonstrated that the concentration of cGMP in the submucosa was four orders of magnitude lower than that required to affect sensory neuron firing (5).

## Neuropods

Enteroendocrine cells (EECs) are scattered throughout the gut mucosa with their apical surface exposed to the lumen. In this orientation, EECs detect and respond to ingested nutrients by secreting hormones from their basal surface into the paracellular space, where the hormones are taken up by blood vessels to reach the systemic circulation. Although originally named for their production of hormones, recently, it has been recognized that EECs possess many neuron-like properties (6). When viewed in three dimensions, cholecystokinin-producing (CCK-producing) and peptide YY–producing (PYY-producing) EECs were found to have basal processes that extended to other cells (7). These processes contained axon-like neurofilaments; mitochondria; and both a large, dense core and small, clear secretory vesicles, consistent with hormone and neurotransmitter vesicles, respectively (8). Moreover, these cells expressed both pre and post-synaptic proteins and were found to synapse with submucosal neurons (9). Based on their neuron-like features, these processes were named neuropods. Following this discovery, enterochromaffin cells, a subtype of EECs that secretes serotonin, have also been shown to synapse with neurons (10). Thus, direct EEC-to-neuron synaptic communication seems to be common to many EEC subtypes. The term neuropod cell has been used to refer to EECs that functionally connect with neurons (9, 11).

## Route for ameliorating visceral pain

Capitalizing on these observations, Barton et al., in the current issue of the *JCI,* reveal how GUCY2C agonists ameliorate visceral pain (12). First, the authors demonstrated that *Gucy2c* was enriched in a subset of intestinal cells possessing neuropods and that these cells expressed both endocrine and neural markers consistent with neuropod cells, thus providing the initial hint that EECs might be a target for GUCY2C agonists. Using intestinal organoids that contained neuropod cells, the authors demonstrated that sensory neurons isolated from dorsal root ganglia grew toward and connected with GUCY2C enriched cells. In this in vitro model, the GUCY2C agonist linaclotide elevated the DRG-neuron rheobase and reduced action potential firing, whereas cGMP administered to the solution bathing organoids and neurons did not affect excitability. Notably, the effects of linaclotide were not seen in *Gucy2c^–/–^* cells. These findings indicated that neuropod cells mediated the linaclotide effects on sensory neurons through a mechanism that could not be reproduced by exogenous cGMP (12).

Importantly, *Gucy2c^–/–^* mice exhibited spontaneous visceral pain that was refractory to linaclotide. The selectivity of this phenomenon for neuropod cells was demonstrated by deletion of *Gucy2c* only in CCK cells. These animals also developed spontaneous visceral pain (12).

Thus, GUCY2C agonists ameliorated visceral pain through neuropod cell signaling (12). Not only is this observation important to our understanding of the pharmacology of GUCY2C agonists, but it provides insights into the pathophysiology of visceral pain. Although it has long been suspected that some disturbance in visceral pain signaling and perception was at the root of IBS, a role for EECs was not evident. The discovery of *Gucy2c* in neuropod cells provided an initial clue, and linking agonist stimulation to a reduction in sensory nerve firing provided mechanistic explanations for both the pain and therapeutic response to GUCY2C-targeted drugs (12). It is interesting to speculate that visceral pain in IBS is due to deficient GUCY2C signaling — either through the lack of sufficient guanylin, and/or uroguanylin (13), or neuropod cell unresponsiveness. These possibilities are important for understanding the fundamental defects underlying unexplained abdominal pain.

Cyclic GMP is the only second messenger signaling pathway known to be activated by GUCY2C. The observation that, in neuropod-connected DRG neurons, action potential firing was suppressed only through GUCY2C activation of neuropod cells and not by exogenous cGMP (12) raises the possibility that cGMP is not the transmitter that reduces DRG firing, and that another, yet unknown, transmitter is involved. Except for L cells that produce both glucagon-like peptides and PYY, when EECs were discovered, the general dictum was that one cell produced one hormone. This concept has since been disproved (14), and it is well accepted that an EEC may express multiple hormones and neurotransmitters (15). In the case of GUCY2C-expressing neuropod cells, identifying this transmitter could unveil another potential target for treating visceral pain.

Although CCK cells are a major EEC type, they represent only a subset of all EECs (16). It is somewhat surprising that deletion of *Gucy2c* only in CCK cells was sufficient to induce spontaneous visceral pain (12). This finding suggests that small changes in neuropod cell GUCY2C signaling can produce pain. Perhaps this mechanism accounts for the large number of individuals afflicted with IBS.

CCK cells are also concentrated in the proximal small intestine. Even though the colon is a site of IBS pain, GUCY2C agonists exert their pain-reducing effects by targeting cells in the small intestine (17, 18). The current study suggests that the neuropod-DRG connection provides an initial link from the proximal intestine to the spinal cord to modulate visceral pain pathways from the distal bowel (12).

## Implications for treatments

GUCY2C agonists ameliorate constipation and IBS pain; however, IBS has multiple manifestations and is commonly associated with diarrhea. Even the same patient may alternate between constipation and diarrhea. Because of their intestinal secretory effects, linaclotide and plecanatide are not used for diarrhea-predominant IBS. It is not known if the pain associated with diarrhea-predominant IBS has the same pathophysiological basis as constipation-predominant IBS, i.e., neuropod GUCY2C–signaling deficiency. However, based on the effects of linaclotide on visceral pain in mice it is tempting to think GUCY2C agonists could reduce pain in IBS regardless of its association with diarrhea or constipation. The discovery by Barton et al. provides a route to dissociate the effects of GUCY2C on pain from intestinal secretion (12). To avoid activating GUCY2C on enterocytes and worsening diarrhea, an attractive strategy to treat visceral pain would be to selectively target neuropod cells.

## Version 1 02/15/2023

Electronic publication
